# Silent Congestion: Chronic Budd-Chiari Syndrome Unmasked by Acute Decompensation

**DOI:** 10.7759/cureus.117029

**Published:** 2026-09-27

**Authors:** Justin McClusky, Jasneet Gill, Chimezie Mbachi

**Affiliations:** 1 Internal Medicine, Community Health Systems - Tennova North Knoxville Medical Center, Knoxville, USA; 2 Internal Medicine, Tennova North Knoxville Medical Center, Knoxville, USA; 3 Gastroenterology and Hepatology, Community Health Systems - Tennova North Knoxville Medical Center, Knoxville, USA

**Keywords:** acute on chronic budd chiari, budd-chiari syndrome, chronic budd-chiari syndrome, cirrhosis, hepatic congestion, myeloproliferative neoplasm (mpn), portal vein hypertension

## Abstract

We report a case of a 27-year-old male patient without previously recognized liver disease who developed progressive abdominal distention, ascites, portal hypertensive physiology, and acute hepatic decompensation due to acute-on-chronic Budd-Chiari syndrome. Multimodality imaging demonstrated recanalization of the umbilical vein, abnormal portal venous flow, and severe hepatic venous outflow obstruction. The case highlights the importance of recognizing chronic imaging findings in patients with acute deterioration and the procedural challenges associated with chronic hepatic venous occlusion.

## Introduction

Budd-Chiari syndrome (BCS) is a rare disorder caused by obstruction of hepatic venous outflow at any level from the small hepatic veins to the inferior vena cava [1]. Its annual incidence is estimated at 0.5-1 per million, with a prevalence of approximately 1-10 per million, although geographic variation exists [2]. The obstruction leads to hepatic congestion, increased sinusoidal pressure, and portal hypertension, which may progress to fibrosis, cirrhosis, and liver failure if left untreated [1,3].

Most cases are associated with underlying prothrombotic conditions, including myeloproliferative disorders and antiphospholipid syndrome, while some remain idiopathic [1,3]. Clinical presentation ranges from mild symptoms such as hepatomegaly and ascites to acute liver failure [4]. Diagnosis relies on clinical suspicion and imaging studies, with invasive testing reserved for selected cases [4].

Acute-on-chronic BCS refers to sudden decompensation in a patient with previously unrecognized or compensated venous outflow obstruction, often triggered by new thrombosis [5]. Acute-on-chronic presentations are relatively uncommon but clinically important because delayed recognition may lead to irreversible hepatic injury [5]. Modern management strategies emphasize early anticoagulation and a stepwise escalation from medical therapy to endovascular intervention and ultimately liver transplantation when necessary [6,7].

## Case presentation

A 27-year-old male patient with no significant past medical history presented with a one-month history of progressive abdominal pain, bloating, distention, nausea, vomiting, and decreased oral intake. His symptoms worsened over the last 1-2 weeks, with abdominal distention becoming more pronounced and eventually culminating in an episode of profuse vomiting, which led to his hospitalization. The patient had a family history of hepatocellular carcinoma (HCC) in both his uncle and grandfather, but no known personal history of liver disease. He reported occasional alcohol use (1-2 beers a month) but denied tobacco and illicit drug use. His prior medical history was unremarkable, aside from intermittent postprandial discomfort years ago.

Upon presentation, his vital signs were relatively stable: blood pressure of 138/92 mmHg, heart rate of 108 bpm, and a temperature of 97.4°F. He was six feet six inches tall, weighed 175 pounds, and had a BMI of 20. Initial lab work revealed some concerning findings: an elevated white blood cell (WBC) count of 11.5 (reference range, 4.0-11.0 × 10³/µL), polycythemia with a hemoglobin level of 18.7 g/dL (13.0-17.0 g/dL), and a significant rise in liver enzymes-aspartate aminotransferase (AST) 320 U/L (10-40 U/L) and alanine aminotransferase (ALT) 593 U/L (7-56 U/L). His total bilirubin was mildly elevated at 2.5 mg/dL (0.1-1.2 mg/dL), with an albumin of 3.0 g/dL (3.5-5.0 g/dL). The patient’s presentation raised concern for acute hepatic dysfunction and possible portal hypertensive physiology.

Imaging studies were performed, starting with a CT scan of the abdomen and pelvis, which showed an enlarged, heterogeneous liver, along with a similarly heterogeneous spleen and moderate ascites. A notable finding seen in Figure 1 was recanalization of the umbilical vein, a classic radiographic marker of chronic portal hypertension and longstanding venous outflow obstruction [8,9]. Additional imaging findings of heterogeneous hepatic enhancement further supported chronic hepatic congestion and impaired venous drainage [9].

**Figure 1:**
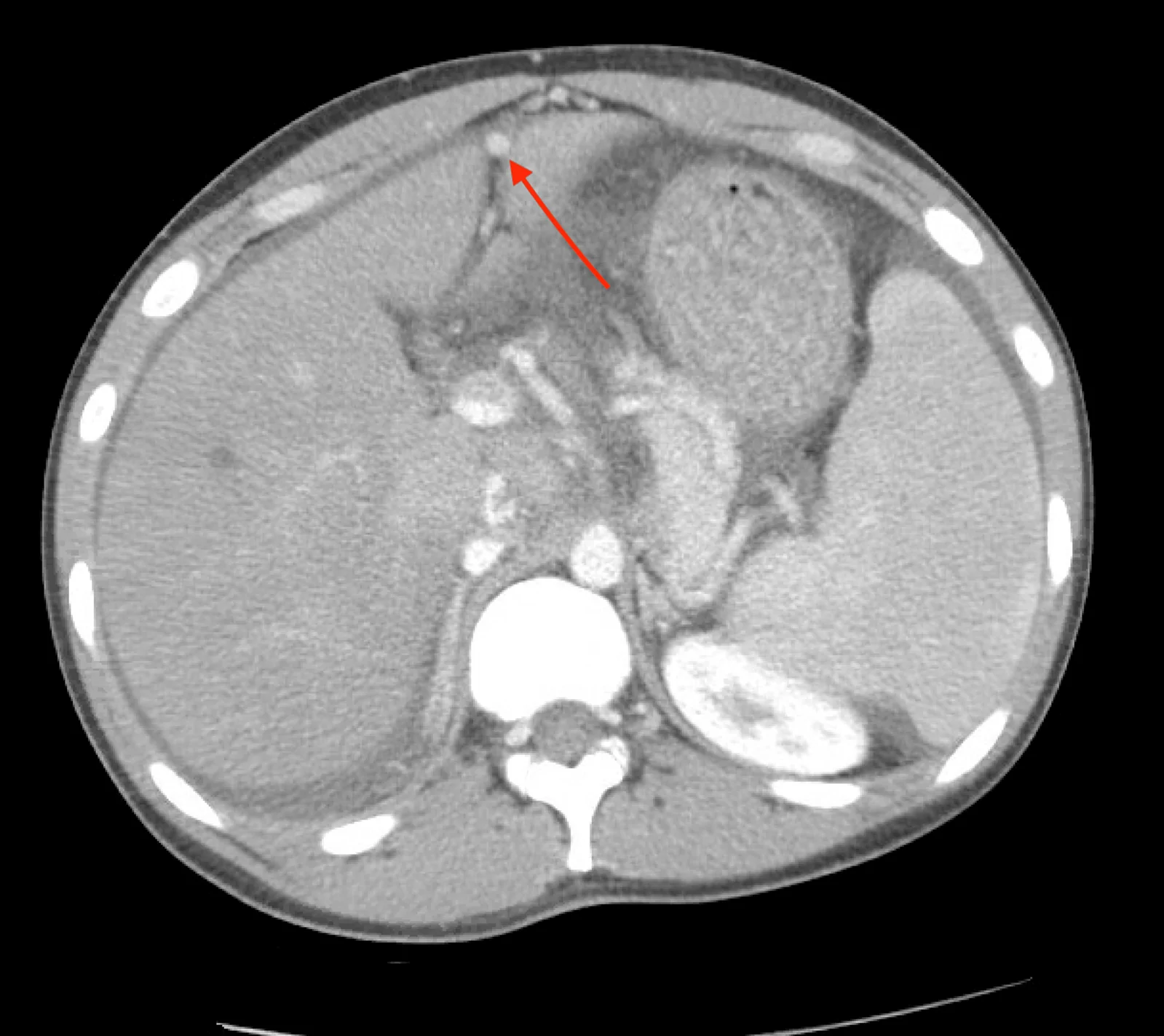
Contrast-enhanced CT of the abdomen demonstrating recanalization of the umbilical vein. The red arrow indicates the recanalized umbilical (paraumbilical) vein, supporting chronic portal hypertension in the setting of Budd-Chiari syndrome.

Figure 2 shows a Doppler ultrasound of the hepatic vessels that revealed low-velocity hepatofugal flow within the main portal vein at the porta hepatis. Figure 3 shows reversed flow in the left hepatic vein. The hepatic artery demonstrated preserved flow, but the overall findings suggested significant hepatic venous outflow obstruction with portal hypertension [4,9].

**Figure 2:**
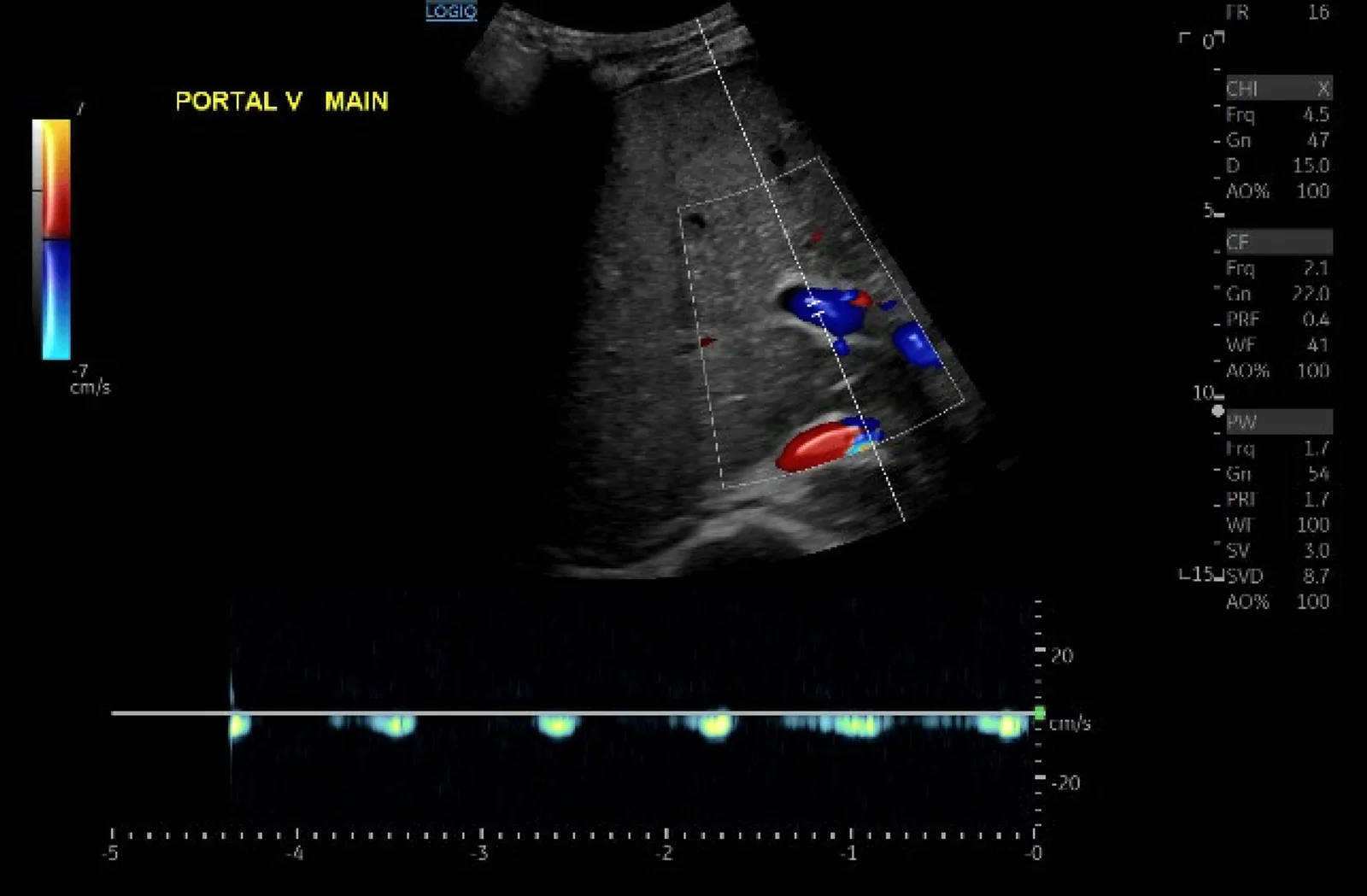
Doppler ultrasound demonstrating hepatofugal flow in the main portal vein. Color and spectral Doppler demonstrate abnormal flow direction within the main portal vein, consistent with portal hypertension.

**Figure 3:**
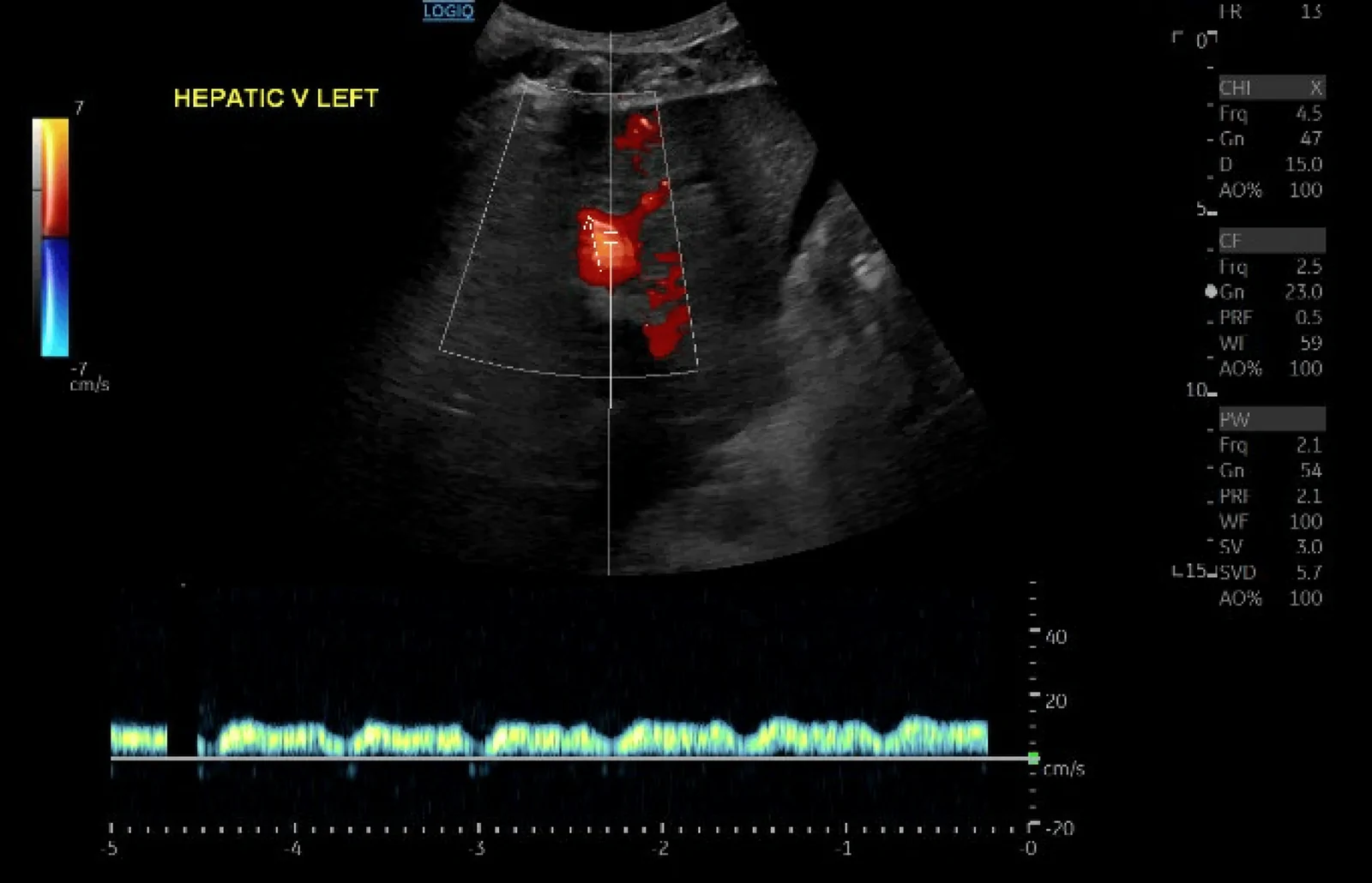
Doppler ultrasound (US) demonstrating reversed flow in the left hepatic vein. US image demonstrating reversal of flow within the left hepatic vein, further supporting abnormal hepatic venous circulation associated with Budd-Chiari syndrome.

A diagnostic paracentesis was performed, removing 2.25 L of ascitic fluid. The analysis revealed a serum-ascites albumin gradient (SAAG) of 3.2, consistent with portal hypertensive ascites, and a total protein concentration of 2.1 g/dL. Cell count showed 1,368 RBCs and 224 WBCs, with 15% neutrophils, and cultures were negative, supporting portal hypertensive ascites.

On the third day of hospitalization, hepatic venography demonstrated absent inflow from the hepatic veins despite a patent inferior vena cava. Multiple attempts to cannulate the hepatic veins were unsuccessful, and no hepatic veins were visualized on cavography. These findings strongly suggested chronic organized hepatic vein thrombosis with fibrotic transformation [8,9].

Regarding his liver function, over the first several days of admission, there was a noticeable trend of worsening liver enzyme levels and coagulation parameters. On hospital day one, AST and ALT were elevated at 320 (10-40 U/L) and 593 U/L (7-56 U/L), respectively, with a total bilirubin of 2.5 mg/dL (0.1-1.2 mg/dL). By hospital day two, AST and ALT decreased slightly to 262 U/L (10-40 U/L) and 510 U/L (7-56 U/L), while bilirubin improved to 2.0 mg/dL. However, by hospital day three, AST and ALT rose sharply to 710 (10-40 U/L) and 881 U/L (7-56 U/L), respectively, with total bilirubin increasing to 2.7 mg/dL (0.1-1.2 mg/dL). Additionally, prothrombin time (PT) and international normalized ratio (INR) progressively worsened, reflecting deteriorating hepatic synthetic function: PT: 23.2 to 30.6 s (reference range, approximately 11-14 s); INR: 2.0 to 2.8 (reference range, 0.8-1.2). These findings were concerning for acute hepatic decompensation secondary to worsening venous outflow obstruction. Given the progressive liver dysfunction and worsening transaminases, the patient was transferred to a tertiary care center for further evaluation, including consideration for direct intrahepatic portosystemic shunt (DIPS) placement or liver transplantation.

## Discussion

BCS results from obstruction of hepatic venous outflow, leading to hepatic congestion, hepatocellular ischemia, portal hypertension, and ultimately hepatic fibrosis or failure if untreated [1,3]. In chronic disease, collateral venous circulation may partially compensate for impaired hepatic outflow and delay symptom onset [3]. Symptoms may not manifest until the thrombotic burden overwhelms collateral compensation.

The pathophysiology of BCS involves increased sinusoidal pressure secondary to impaired venous drainage, causing hepatic congestion and hypoxic injury [1]. Over time, chronic venous obstruction promotes fibrosis and cirrhosis [3]. In this patient, imaging findings including recanalization of the umbilical vein, heterogeneous hepatic enhancement, and inability to visualize or cannulate the hepatic veins strongly suggested longstanding disease with collateral vessel formation [8,9].

Diagnosis of BCS relies heavily on imaging studies. Doppler ultrasound is frequently the first diagnostic modality and may demonstrate absent or reversed portal venous flow [4,9]. CT and MRI provide further characterization of hepatic morphology, collateral circulation, and thrombosis [9]. Characteristic radiographic findings of chronic BCS include caudate lobe hypertrophy, heterogeneous hepatic enhancement, intrahepatic collateralization, and recanalization of paraumbilical veins [9]. Venography remains the gold standard for definitive evaluation of hepatic venous anatomy and procedural planning [4].

In this case, interventional radiology was unable to cannulate the hepatic veins, suggesting chronic organized thrombosis and fibrotic cord transformation [8,9]. This finding has important therapeutic implications. Chronic fibrotic occlusion markedly reduces the likelihood of successful thrombolysis or mechanical thrombectomy [6,8]. Furthermore, conventional transjugular intrahepatic portosystemic shunt (TIPS) placement was not technically feasible because hepatic venous access could not be established [6]. As a result, transfer for possible DIPS, which creates a shunt directly from the inferior vena cava to the portal venous system, became necessary [6].

Endovascular interventions such as angioplasty, TIPS, and DIPS have significantly improved outcomes in patients with BCS, particularly when performed before irreversible hepatic injury develops [6]. TIPS has emerged as a preferred intervention for patients who fail medical therapy or present with progressive portal hypertension and hepatic dysfunction [6]. However, chronic complete hepatic vein occlusion may prevent successful TIPS placement, requiring alternative approaches such as DIPS or liver transplantation [6].

A comprehensive thrombophilia evaluation is essential in young patients presenting with BCS [1,2]. Common associated disorders include JAK2-positive myeloproliferative neoplasms, antiphospholipid syndrome, factor V Leiden mutation, protein C or S deficiency, antithrombin deficiency, and paroxysmal nocturnal hemoglobinuria [2,5]. The patient’s elevated hemoglobin raised concern for an underlying myeloproliferative disorder, and hematologic evaluation was initiated. Recent literature has emphasized that myeloproliferative disorders remain the most common identifiable cause of BCS in Western populations [7]. Identification of an underlying prothrombotic condition in patients with BCS may influence the duration of anticoagulation, assessment of recurrent thrombosis risk, and long-term management.

Historically, BCS carried a poor prognosis, with high mortality rates in untreated patients. However, advances in anticoagulation, interventional radiology, and liver transplantation have substantially improved long-term survival [10]. Current management follows a stepwise treatment approach beginning with anticoagulation, followed by angioplasty or shunting procedures, and ultimately liver transplantation for patients with fulminant liver failure or refractory portal hypertension [4,6,7]. Studies have demonstrated that timely intervention and restoration of hepatic outflow are strongly associated with improved transplant-free survival [10].

## Conclusions

We present a case of acute-on-chronic BCS in a young male without previously recognized liver disease who developed progressive portal hypertension and acute hepatic decompensation. This case highlights several important teaching points: first, chronic BCS may remain clinically silent until collateral circulation fails; second, imaging findings such as umbilical vein recanalization and inability to cannulate hepatic veins strongly suggest chronic organized thrombosis; and third, chronic fibrotic hepatic vein occlusion may preclude conventional TIPS placement, necessitating advanced interventions such as DIPS or liver transplantation evaluation. Early recognition, comprehensive thrombophilia evaluation, prompt anticoagulation, and timely referral to specialized centers remain essential for improving outcomes in patients with BCS.
